# Application of 3D technology for the design of a guidewire for deep cervicofacial collections percutaneous management

**DOI:** 10.4317/jced.61354

**Published:** 2024-03-01

**Authors:** Clara López-Martínez, Marta-María Pampín-Martínez, Alba García-López-Chicharro, Íñigo Aragón-Niño, José-Luis Cebrián-Carretero

**Affiliations:** 1 Oral and Maxillofacial Surgery Department, University Hospital La Paz, Madrid, Spain

## Abstract

**Background:**

This study aims to present a case of deep cervicofacial abscesses and demonstrate the efficacy of CT-guided drainage with a custom-designed puncture guide in a challenging anatomical location. The use of this type of guide is an innovative technique since CAD-CAM technology has not been used for this type of application until now.

**Material and Methods:**

A 76-year-old man with persistent facial swelling and trismus underwent surgical drainage initially, but symptoms persisted. A CT-guided transcutaneous approach was planned using a custom-designed positioning guide created with “in-house” 3D technology. The guide was fabricated using Surgical Guide resin, and the patient underwent successful CT-guided drainage.

**Results:**

The intervention facilitated precise drainage without damaging critical anatomical structures. The patient exhibited prompt clinical improvement, shortened hospitalization, and favorable aesthetic outcomes.

**Conclusions:**

CT-guided drainage, particularly when combined with a custom puncture guide, offers a less invasive alternative for challenging cervicofacial abscesses. This approach proves valuable in reducing procedure duration, minimizing soft tissue trauma, and enhancing preoperative planning, making it especially beneficial for patients with high anesthetic risk or complex anatomical considerations.

** Key words:**Cervical abscess, 3D technology, drainage guide, percutaneous puncture.

## Introduction

The prevalence of cervicofacial abscesses is decreasing thanks to the widespread use of antibiotics. However, they are a common pathology in hospital clinical practice. The characteristics and clinical findings will vary depending on the cervical space or spaces involved. The most frequent clinical findings are pain, facial swelling, trismus, odynophagia, dysphagia, otalgia, fever, etc (1).

The use of a CT scan, especially when endovenous contrast is used, is a useful tool for evaluation and diagnosis, especially when identifying the area that the abscess occupies.

The therapeutic management of these collections includes, on many occasions, surgical or percutaneous drainage together with appropriate and early antibiotic treatment (2).

On certain occasions, these abscesses can be located in complex or difficult-to-reach anatomical regions such as the pterygopalatine or infratemporal fossa. It is in these cases when image-guided drainage techniques can be useful to solve the infection, minimizing morbidity compared to open surgical drainage, which could produce serious side effects (nerve or great vessel damage, for example).

This paper describes the case of a 76-year-old man who was referred to the emergency department of the University Hospital La Paz with right-side facial swelling which began 7 days ago. Upon clinical examination, there was right-side facial swelling with hardness, associated with a 20 mm trismus. The requested CT scan showed two abscesses in the deep medial pterygoid and right masseteric space. Initially, drainage of the collections was performed under general anesthesia through an intraoral approach in the region of the right maxillary tuberosity and ipsilateral jugal mucosa, obtaining outflow of purulent material. Also, extraction of the right first upper molar was performed. However, the patient’s symptoms did not improve within 12 days of admission, with the persistence of facial swelling and trismus. Blood tests indicated the subsistence of an active infection. A postoperative CT scan was performed which revealed the persistence of the abscess cavity at the medial pterygoid space. Therefore, the decision to re-operate was made. Given the size and location of the cavity, a CT-guided transcutaneous approach was planned. A positioning guide was virtually designed, using 3D technology, to direct the puncture towards the collection, thus avoiding damage to relevant anatomical structures such as the condyle and the mandibular ramus.

## Case Report

First, the segmentation of the skin, the jaw and the abscess to be drained is performed from the DICOM file using Brainlab iPlan CMF software. In this case, for the segmentation of the mandible, the auto segmentation tool may be used. It is important to segment the mandible to ensure that there is no interference of the mandible with the puncture path. Whereas, for the segmentation of the collections the “Smart brush” tool is used, performing the segmentation manually. For the segmentation of the skin, the “Threshold” tool is used.

**Figure 1:**
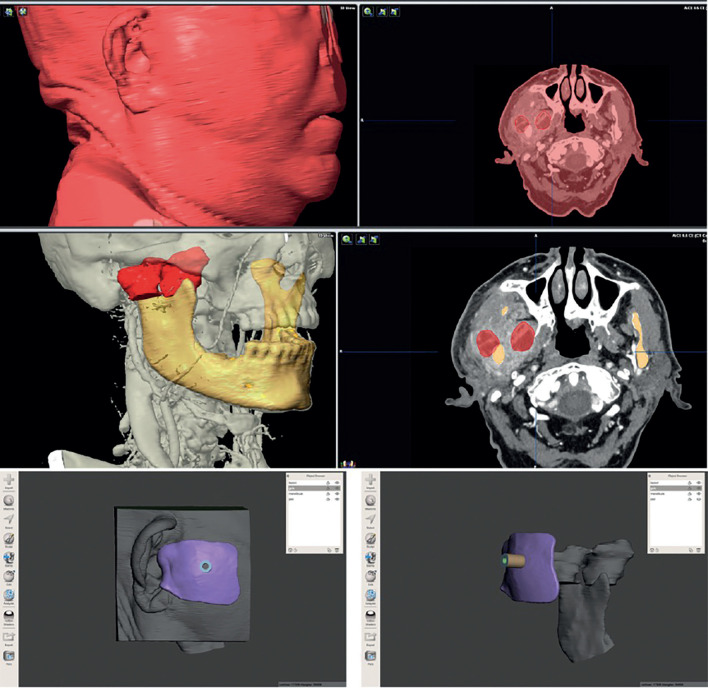
Segmentation of the skin, the jaw and the abscess using Brainlab iPlan CMF software and design of the guide using the tragus as a fixed reference to position it.

Subsequently, the 3 objects are exported to the Meshmixer software as Standard Tessellation Language (STL) files. Afterwards, the “Select” tool is used to select the region of the skin on which the guide should be placed, using the tragus as a fixed reference to position it. With the “Offset” tool a sheet is created 0.3 mm away from the skin, and with the “Separate shells” tool it is separated from the skin STL. With the “Offset” tool another sheet is created 3 mm away from the previous one connected to it, giving as a result the positioning guide. Then, a cylinder is added and, with the “Transform” tool, it is given the desired size and orientation, making it head towards both collections avoiding the jaw. Using the “Duplicate” tool we duplicate this cylinder with a smaller thickness and a longer length using the “Transform” tool. The thick cylinder is selected and the union with the guide is created using the “Boolean union” tool. After that, the thinner cylinder is selected and deducted from the rest of the complex using the “Boolean difference” tool. Thus, the positioning guide is obtained together with the cylinder that will orient the puncture needle (Fig. 1).

Finally, the STL file is exported and printed with the Formlabs printer using biocompatible “Surgical Guide” resin. At the impression time, it is important to place the guide so that the bearing area on the patient is facing upwards, in order to avoid the formation of impression abutments on this side. In addition, the abutments formed on the inside of the cylinder must be removed so that the guide is not obstructed. After printing, the models are washed for 10 minutes in a Form Wash with 99% isopropyl alcohol and cured at 60°C for 30 minutes in a Form Cure.

## Results

**Figure 2:**
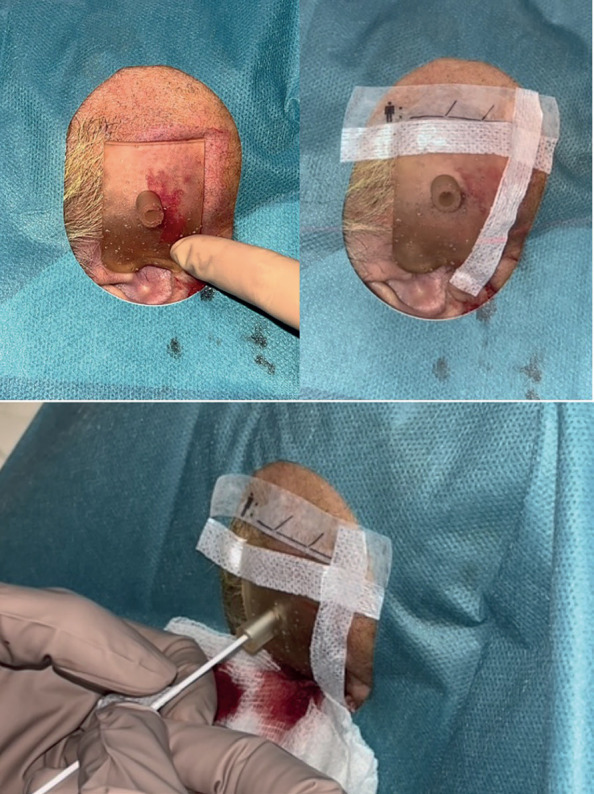
Drainage guide placement on the patient and attachment with sterile adhesive. Percutaneous drainage under local anesthesia through the designed guidewire.

**Figure 3:**
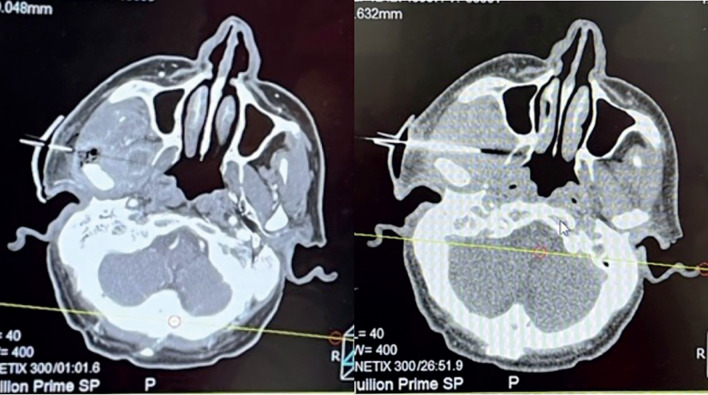
CT image during puncture needle insertion prior to collection drainage and CT image after collection drainage.

The guide was positioned over the patient, which engaged well with the tragus but for further fixation, sterile adhesives were used. The puncture to drain the abscess was performed by the radiology team using CT guidance (Fig. 2). The cavity was successfully drained, showing a reduction in size before and after the procedure (Fig. 3).

The intervention performed allowed drainage of the collections without damaging any relevant anatomical structure. Therefore, the patient showed prompt clinical, analytical, and radiological improvement and was discharged from the hospital two days after the procedure.

## Discussion

The first two cases of CT-guided abscess drainage were described by Cole *et al*. in 1984 (3). Poe *et al*. described CT-guided aspiration in four patients with complicated cervical abscesses in 1996 (4).

CT-guided abscess puncture is a far less invasive technique than surgical drainage, and neither requires general anesthesia: local anesthesia at the puncture site is enough. Since percutaneous puncture is a minimally invasive procedure, hospitalization time is shortened and the aesthetic results are better, since skin scars are avoided (5). In such anatomically complex locations, it allows the drainage procedure to be performed with less morbidity for the patient. Moreover, in these cases, in which the collections are located in deep cervicofacial spaces such as the pterygomandibular space, ultrasound loses utility, due to the interference of hard tissues that prevent a correct visualization of the collections.

Furthermore, the use of a guide to direct the puncture helps to perform the drainage of the collection without the repeated puncturing of the patient and the requirement to reroute the needle. It has an advantage over navigation since it does not require the placement of a navigation reference on the skull and can be performed under local anesthesia. Moreover, it will not only reduce the duration of the procedure compared to CT-guided puncture without the use of guides, but also reduce the soft tissue trauma. In addition, the required preoperative planning of the guidewire design will provide a better understanding of the patient’s anatomy and the location of the abscess. As drawbacks, it requires several CT image sequences to corroborate proper localization of the drainage needle, and there may also exists a certain degree of inaccuracy in the locating power and poor support of the guidewire when placed on soft tissues.

## Conclusions

In conclusion, CT-guided drainage of deep cervicofacial abscesses is an efficient alternative in patients with high anesthetic risk, and in abscesses located in deep spaces difficult to access for surgical intraoral drainage or small cavities. In addition, the authors believe that the combination of this technique with a puncture guide will improve its effectiveness and reduce tissue damage.

## References

[1] Almuqamam M, Gonzalez FJ, Kondamudi NP (2022). Deep Neck Infections. In: StatPearls. Treasure Island.

[2] Bhardwaj R, Makkar S, Gupta A, Khandelwal K, Nathan K, Basu C (2022). Deep Neck Space Infections: Current Trends and Intricacies of Management?. Indian J Otolaryngol Head Neck Surg.

[3] Cole DR, Bankoff M, Carter BL (1984). Percutaneous catheter drainage of deep neck infections guided by CT. Radiology.

[4] Poe LB, Petro GR, Matta I (1996). Percutaneous CT-guided aspiration of deep neck abscesses. AJNR Am J Neuroradiol.

[5] Thanos L, Mylona S, Kalioras V, Pomoni M, Batakis N (2005). Potentially life-threatening neck abscesses: therapeutic management under CT-guided drainage. Cardiovasc Intervent Radiol.

